# Special Issue: Microbial Impact on Cholesterol and Bile Acid Metabolism

**DOI:** 10.3390/microorganisms10020477

**Published:** 2022-02-21

**Authors:** Jason M. Ridlon

**Affiliations:** 1 Department of Animal Sciences, University of Illinois at Urbana-Champaign, Urbana, IL 61801, USA; jmridlon@illinois.edu; 2 Carl R. Woese Institute for Genomic Biology, University of Illinois at Urbana-Champaign, Urbana, IL 61801, USA; 3 Division of Nutritional Sciences, University of Illinois at Urbana-Champaign, Urbana, IL 61801, USA; 4 Cancer Center at Illinois, University of Illinois at Urbana-Champaign, Urbana, IL 61801, USA; 5 Center for Advanced Study, University of Illinois at Urbana-Champaign, Urbana, IL 61801, USA

An important current focus in microbiome science is the biotransformation and degradation of cholesterol, steroids, and bile acids [1]. In vertebrates, cholesterol is the precursor to all steroids, including bile salts. Roughly 1g of cholesterol is excreted into feces daily, originating from a combination of diet, bile, and sloughed host cells. Biotransformations of cholesterol by anaerobic gut bacteria yields a non-absorbable product known as coprostanol. Coprostanoligenic bacteria have important potential to regulate cholesterol levels in the blood and, thus, impact cardiovascular health [2]. Progress in this area has been slow, due largely to the great difficulty working with coprostanoligenic bacteria. An excellent review in this Special Issue by Catherine Juste and Philippe Gérard, entitled “Cholesterol-to-Coprostanol Conversion by the Gut Microbiota: What We Know, Suspect, and Ignore”, provides historical analysis and critical evaluation of current knowledge in microbial cholesterol metabolism by leading experts [3].

Bile acid synthesis is a major route of cholesterol removal from the body. The multistep conversion of cholesterol to bile acids in the liver removes 500–800 mg cholesterol from the body daily. The daily amount of bile acid synthesis maintains the bile salt pool in a steady state, coinciding with the amount of bile salts that escape enterohepatic circulation and are excreted in feces [4]. The metabolism of bile salts begins with a “gateway reaction” in which conjugated bile salts are deconjugated by bile salt hydrolase enzymes releasing either taurine or glycine and unconjugated primary or secondary bile acids [5]. Two articles focus on gut microbial bile salt hydrolase enzymes. A primary research article by Déjean et al. entitled, “Identifying a Novel Bile Salt Hydrolase from the Keystone Gut Bacterium *Christensenella minuta*” describes BSH activity and phylogenetic position of the *C. minuta* enzyme [6]. An expert review by Daly et al. entitled, “Functional and Phylogenetic Diversity of BSH and PVA Enzymes” places BSH enzymes in their wider phylogenetic context of the choloylglycine hydrolase family, which also includes penicillin V acylases [7]. Further diversification of bile salts includes the interconversion of steroid ring hydroxyl groups from ɑ-hydroxyl ↔ oxo-group ↔ β-hydroxyl by microbial hydroxysteroid dehydrogenases (HSDHs) [8,9]. Recent advances in understanding of microbial HSDHs is presented by Doden and Ridlon in “Microbial Hydroxysteroid Dehydrogenases: From Alpha to Omega” [10].

Bile salts are important nutrient signaling hormones, and the physiological and pathophysiological effects of the diversification of bile salts by host-associated bacteria are becoming established and accumulating [11]. Kiriyama and Nochi provide a detailed review of the formation of hydrophobic secondary bile acids, the signaling pathways activated by bile acids, and the implications for host health in their article, “Physiological Role of Bile Acids Modified by the Gut Microbiome” [12]. The diversification of bile salts by bacteria also shapes the structure and function of host-associated microbes. In their article, “Functional Characterisation of Bile Metagenome: Study of Metagenomic Dark Matter”, Sabater et al. provide potentially novel enzymes involved in tolerance to the high bile salt concentrations in the gallbladder of humans [13]. By contrast, the use of bile acid binding resins to reduce the bioavailability of bile salts offer therapeutic options [14]. In their article, “Bile Acid Sequestrant, Sevelamer Ameliorates Hepatic Fibrosis with Reduced Overload of Endogenous Lipopolysaccharide in Experimental Nonalcoholic Steatohepatitis”, Tsuji et al. provide evidence that the bile acid sequestrant, sevelamer, reduced bile acid absorption and increased LPS excretion, inhibiting the development of murine hepatic steatohepatitis [15].

Bile salt metabolism extends far beyond the gastrointestinal tract of vertebrates. The American microbiologist, Stanley Falkow, is said to have been fond of telling his students, “The world is covered in a fine patina of feces” [16]. This is certainly true of our collective agricultural efforts. Manuring soils creates a rich source of carbon, including bile salts, and it is thus no surprise that microbes have evolved complex biochemical pathways to mineralize bile salts to CO_2_. One consequence of this process is the formation of endocrine-disrupting steroid intermediates that negatively affect soil nematodes [17]. Bodo Philipp’s research group organizes the complexity of bile salt mineralization by environmental microbes into a highly accessible review entitled, “Degradation of Bile Acids by Soil and Water Bacteria” [18].

Altogether, this Special Issue represents valuable expertise and insights from across the world on important topics in the field of cholesterol and bile salt metabolism by host-associated and environmental microbes, and the consequences thereof.

## Data Availability

Not applicable.
